# Endoscopic ultrasound-guided embolization: Successful management of hemorrhage from gastric artery aneurysm rupture

**DOI:** 10.1055/a-2598-5371

**Published:** 2025-05-28

**Authors:** Beinan Hu, Guilian Cheng, Zhenyun Gong, Duanmin Hu

**Affiliations:** 1 105860 Department of Gastroenterology, Second Affiliated Hospital of Soochow University, Suzhou, China

Visceral artery aneurysms (VAAs), though uncommon in clinical practice, pose a critical threat due to their life-threatening rupture. We present a case of a ruptured aneurysm arising from a branch of the right gastric artery. Hemorrhage was managed with endoscopic ultrasound (EUS)-guided embolization.

A 69-year-old man was transferred to our department due to recurrent melena for 5 days. The patient had been administered meloxicam for 3 weeks to manage lumbar disc herniation and had not undergone gastroscopy before. Initial contrast-enhanced CT was performed to evaluate the potential for hemorrhage related to gastrointestinal malignancies. A hyperdense spherical lesion was identified on CT ( Fig. 1 ). Subsequent three-dimensional reconstruction demonstrated a saccular structure ( Fig. 2 ), confirming the diagnosis of an aneurysm. Then, the patient received an endoscopy for hemostasis and further examination. Gastroscopy was compromised by active hemorrhage, and EUS and Doppler demonstrated an aneurysm with active bleeding and exhibiting direct continuity with extramural vessels. Spectral Doppler analysis revealed arterial waveforms. Based on the correlative findings from CT and Doppler waveform analysis, considering the aneurysm was arising from a branch of the right gastric artery along the lesser curvature. 1 ml n-Butyl-2-Cyanoacrylate was injected into the aneurysm lumen under EUS guidance, and Doppler confirmed blood flow arrest ( Video 1 ), achieving primary hemostasis. CT performed 3 days after treatment revealed no symptoms of residual perfusion ( Fig. 3 ). No rebleeding episodes or procedure-related complications were observed during the 1-month follow-up.

**Fig. 1:**
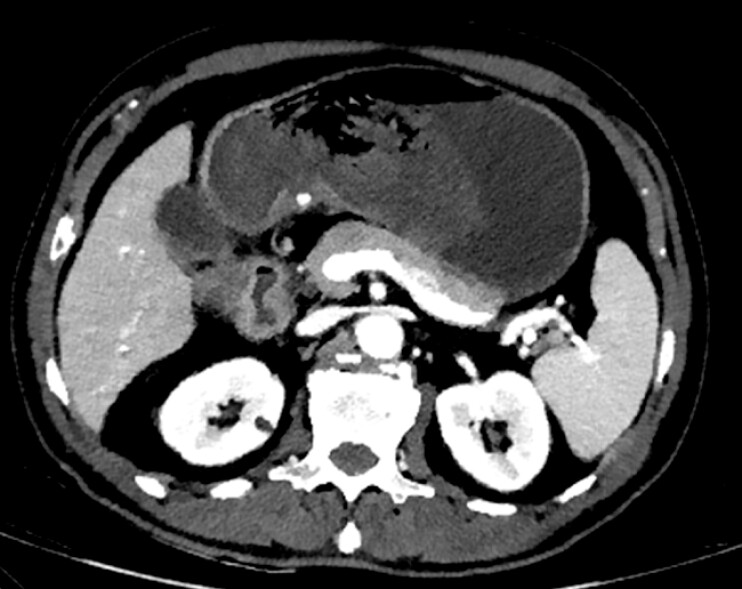
CT image of the hyperdense spherical lesion located in the lesser curvature.

**Fig. 2:**
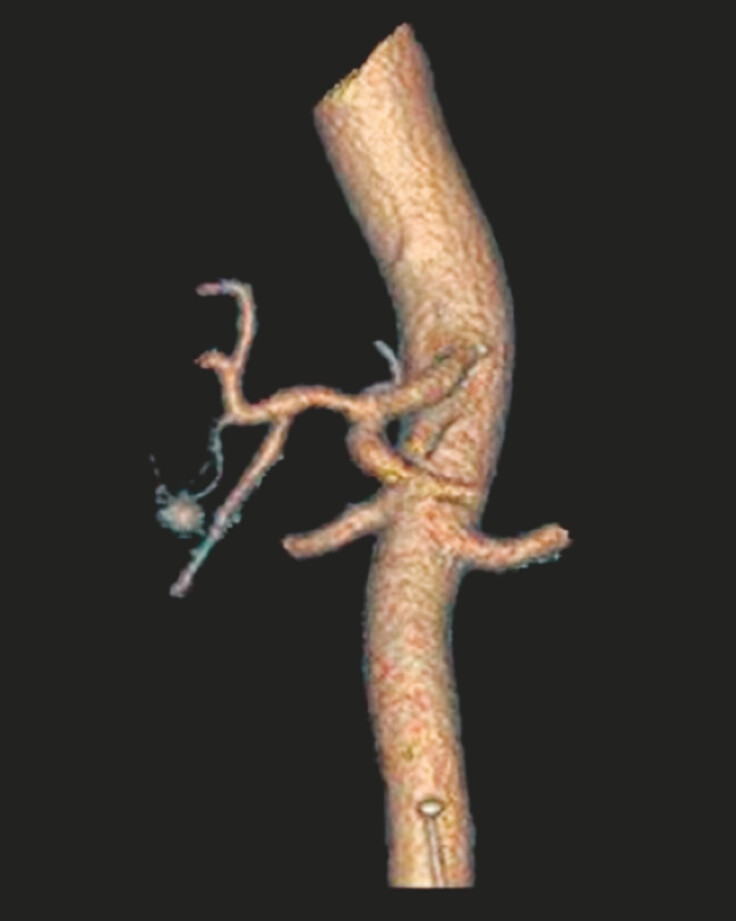
Three-dimensional reconstruction showed a saccular structure.

**Fig. 3:**
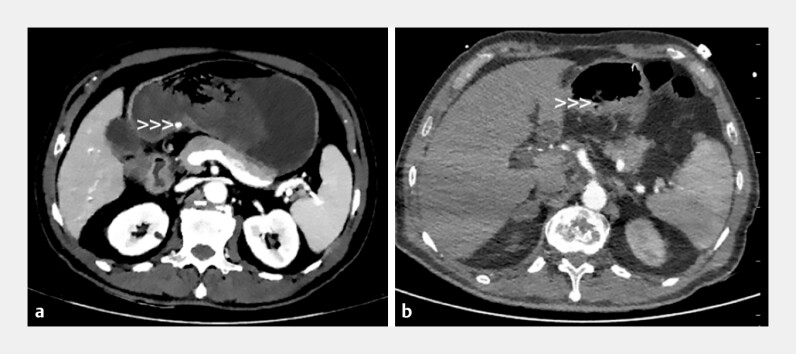
CT images before and 3 days after treatment: **a** CT image before treatment. **b** CT image 3 days after treatment.

Procedure of the embolization under the guidance of EUS.Video 1

VAAs are rare with incidence rates ranging from 0.01 to 0.2% 1 and 25% of VAAs can present with life-threatening rupture 2 . Current management of ruptured VAAs faces technical limitations in superselective embolization and elevated perioperative risks associated with emergency laparotomy. EUS-guided embolization achieved satisfactory results, circumvented parent vessel occlusion risks and invasive surgery. Our experience suggests EUS-guided embolization may serve as an effective alternative for digestive tract-adjacent aneurysms. Further multicenter studies are warranted to validate its safety profile.

Endoscopy_UCTN_Code_TTT_1AO_2AD

## Notes

Endoscopy E-Videos https://eref.thieme.de/e-videos
*E-Videos* is an open access online section of the journal *Endoscopy* , reporting on interesting cases and new techniques in gastroenterological endoscopy. All papers include a high-quality video and are published with a Creative Commons CC-BY license. Endoscopy E-Videos qualify for HINARI discounts and waivers and eligibility is automatically checked during the submission process. We grant 100% waivers to articles whose corresponding authors are based in Group A countries and 50% waivers to those who are based in Group B countries as classified by Research4Life (see: https://www.research4life.org/access/eligibility/ ). This section has its own submission website at https://mc.manuscriptcentral.com/e-videos .
